# Optical Attenuation Coefficient-Based En Face Optical Coherence Tomography Imaging for the Reliable Assessment of the Ellipsoid Zone

**DOI:** 10.3390/jcm13237140

**Published:** 2024-11-25

**Authors:** Hiroaki Sakai, Riku Kuji, Yoshikiyo Moriguchi, Shoko Yamashita, Ayako Takamori, Masato Tamura, Toshihiro Mino, Masahiro Akiba, Hiroshi Enaida

**Affiliations:** 1 Department of Ophthalmology, Faculty of Medicine, Saga University, 5-1-1 Nabeshima, Saga 849-8501, Japan; 2 Research & Development Division, Topcon Corporation, 75-1 Hasunuma-cho, Itabashi-ku, Tokyo 174-8580, Japanymoriguchi@topcon.com (Y.M.); makiba@topcon.com (M.A.); 3 Clinical Research Center, Saga University Hospital, 5-1-1 Nabeshima, Saga 849-8501, Japan

**Keywords:** optical attenuation coefficient, en face, ellipsoid zone, SS-OCT, structural integrity

## Abstract

**Objective:** This study used optical attenuation coefficient (OAC)-based optical coherence tomography (OCT) en face images to assess the ellipsoid zone (EZ) in the foveal region. **Methods:** This retrospective, observational, cross-sectional study of 41 healthy volunteers and 34 patients with retinal diseases included imaging data acquired using a prototype swept-source OCT system. EZ en face images were generated from OCT raster scan volumes based on OAC, followed by denoising and binarization to quantify the percentage of EZ structural normality or abnormality relative to the total imaging area. We evaluated the reliability of the OAC-based method compared with the OCT signal intensity-based method in healthy and diseased eyes. In addition, the validated program was applied to patients with various retinal conditions. **Results:** The estimated normal EZ area in healthy eyes was 96.2 ± 5.6% using the OAC-based method versus 89.3 ± 18.8% for the intensity-based method. The OAC-based method effectively mitigated various artifacts caused by retinal blood vessels and other factors in both healthy and diseased eyes. In a pilot study involving six diseased eyes, the area exhibiting EZ structural abnormalities was 27.5–99.6%. **Conclusions:** The OAC-based EZ assessment robustly suppressed image artifacts and reliably characterized structural abnormalities in the EZ from OCT volumes.

## 1. Introduction

In optical coherence tomography (OCT), metrics reflecting tissue optical properties, such as signal intensity, have gained prominence as biomarkers complementing traditional structural parameters such as layer thickness and lesion size [1,2,3,4,5]. In particular, the signal intensity of the ellipsoid zone (EZ) hyper-reflective band—primarily sourced from photoreceptor mitochondria—could represent a surrogate marker of photoreceptor health, reflecting metabolic functions, structural integrity, and photoreceptor density [4,6].

Despite the utility of OCT signal intensity for assessing EZ-related pathologies in retinal diseases [7,8,9,10], several challenges persist. Macular photoreceptors, with dimensions of approximately 2 μm [11], are substantially smaller than the lateral resolution of conventional swept-source (SS)-OCT (approximately 15–20 μm), potentially compromising the accurate delineation of the EZ states. Issues such as speckle noise, projection artifacts, and motion artifacts complicate EZ assessment, making OCT signal intensity-based assessment less specific for characterizing distinct retinal layers.

We previously enhanced a commercial SS-OCT system by increasing the A-scan speed to 400 kHz, improving the lateral resolution to 5 μm, and implementing a variable cross-cylinder (VCC) lens to correct lower-order aberrations [12]. Herein, we developed a novel image processing procedure that utilizes the optical attenuation coefficient (OAC)—calculated from raw OCT data—to analyze the EZ condition in the en face plane. The OAC-based method measures light scattering within retinal tissue, potentially providing more tissue-specific characterization while reducing projection artifacts. Because it is based on a physical quantity, this approach aims to standardize metrics across different imaging systems. Previous studies have highlighted the promise of OAC-based techniques for assessing retinal pigment epithelium deficits in dry age-related macular degeneration [5,13]. However, OAC-based methods have other potential applications that provide valuable insights into other retinal layers, including the EZ band.

Herein, we evaluated the reliability of the OAC-based EZ evaluation procedure—the OAC-based method or OAC(+)—in comparison with standard OCT intensity-based evaluation—the intensity-based method or OAC(−)—in healthy and diseased eyes using a high-speed, high-resolution SS-OCT. Additionally, as a pilot investigation, we explored the suitability of our OAC-based method across different diseases.

## 2. Materials and Methods

### 2.1. Participants

Healthy volunteers (41 eyes) were recruited from the student population of Saga University Faculty of Medicine. Healthy participants with a history of eye disease or ocular surgery were excluded. Additionally, 34 patients (34 eyes) with various retinal disorders were recruited from Saga University Hospital. These included 25 eyes with operated (previously fovea-off) retinal detachment, 4 eyes with central serous chorioretinopathy, and 1 eye each with Bietti’s crystalline chorioretinal dystrophy, rod–cone dystrophy, operated macular hole, occult macular dystrophy, and diabetic retinopathy. All participants underwent comprehensive ophthalmologic examinations, including refraction testing, best-corrected visual acuity testing, slit-lamp examination, fundus examination, and color fundus photography, in addition to SS-OCT. In cases with retinal disorders, further examinations were required; we performed fluorescein angiography and multifocal ERG as necessary. For both retinal detachment and macular hole cases, the postoperative eyes were examined. Among patients with retinal disease, eyes with significant absorptive materials, such as severe retinal edema, retinal hemorrhage, or intraretinal/subretinal fluid, were excluded from the measurements because these absorbers hindered EZ evaluation.

### 2.2. Clinical Evaluations

All participants underwent comprehensive ophthalmologic examinations and SS-OCT between November 2021 and January 2024. Examinations included best-corrected visual acuity (BCVA) testing, slit-lamp examination, fundus examination, color fundus photography, and refraction testing.

### 2.3. Image Acquisition

A high-speed, high-resolution SS-OCT prototype system (Topcon Corp., Tokyo, Japan) was used. The system featured a wavelength-tunable laser at a scanning rate of 400 kHz in a 1-μm wavelength range and achieved lateral and axial resolutions of approximately 5.5 and 8.8 μm (full width at half maximum), respectively. Fovea-containing three-dimensional OCT data (1.5 × 1.5 mm^2^, 512 × 512 pixels) were acquired within 1.2 s. The system incorporated a VCC lens to correct lower-order ocular aberrations [12]. To visualize the minute structures of the human retina without pupil dilation, the OCT beam diameter was set to 3 mm at the pupil, and the probe beam power was 1.85 mW, adhering to laser safety standards IEC 60825-1:2014 and ANSI Z80.36-2021. SS-OCT data were postprocessed using an embedded custom OCT signal processing software (OACTool, ver.1.0).

### 2.4. Image Analysis

**Figure 1:**
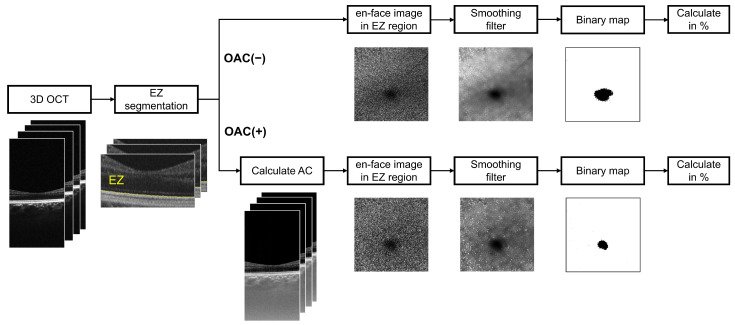
Data processing workflow for quantifying structural abnormalities in the EZ from OCT volumes with and without OAC. En face images of the EZ band were generated from the OCT volume using the OAC-based method (OAC(+)) and the intensity-based method (OAC(−)). These en face images were then binarized to assess the proportion of normal or abnormal region within the imaged area. The black areas in the binarized images represent EZ impairments, ranging from moderate to complete loss.

We evaluated en face images generated using OAC(+) in comparison with OAC(−). The detailed image processing pipeline from OCT volume acquisition to binarized en face image quantification is illustrated in Figure 1, which also highlights the differences between the two methods. For OAC-based en face image generation, the depth-dependent OAC value (***μ***) was computed using the following equation [14]:

(1) $$\left(i\right)=\frac{1}{2\Delta }log\left(1+\frac{I(i)}{\sum_{i+1}^{\infty }I\left(i\right)}\right),$$

where ***i*** is the ***i***’th pixel along an A-scan, ***I***(***i***) is the linear-scale OCT signal intensity at the depth ***i***, and **Δ** is the pixel size.

To assess the photoreceptor status, the EZ slab was extracted from OCT volumes via automated segmentation, with the EZ band defined as the region from the uppermost boundary of the EZ band to 15.6 μm below this boundary considering a foveal EZ thickness of 24.1 ± 0.5 μm [15]. The segmentation results were reviewed by a retina specialist (HS), and manual corrections were performed as required. The en face image was generated by averaging ***μ*** in the z-direction of the EZ slab. A nonlocal mean filter was then applied to the en face image to reduce speckle noise while preserving fine structural details [16] with the filtering intensity parameter ***H*** = 24. Next, the image was binarized using the Phansalkar method, which differentiates between hyper- and hypo-reflective EZ areas through adaptive thresholds [17,18,19]. Using the Phansalkar method, the threshold in a window radius ***R*** (pixel) was computed as

(2) $$threshold=mean(\left(1+p\cdot e^{-q\cdot mean}+k\cdot (\frac{std}{r}-1)\right)),$$

where “***mean***” and “***std***” denote the local mean and standard deviation within radius ***R***, respectively, and ***p***, ***q***, ***r***, and ***k*** are weighting parameters. The specific parameters were ***R*** = 13 and ***k*** = 0.475 for OAC(+) and ***R*** = 13 and ***k*** = 1.0 for OAC(−), whereas ***p*** = 3, ***q*** = 10, and ***r*** = 0.5 were set to their default values. ***R*** was selected to accurately reflect fine structures associated with diseases, whereas ***k*** was adjusted to maintain consistency between images before and after binarization using typical cases. Consequently, the hypo- and hyper-reflective areas in the EZ en face image were colored black and white, respectively, to indicate abnormal and normal states of the EZ. All procedures were implemented using Python (version 3.6). The effectiveness of these procedures was evaluated by comparing the percentage of black or white areas relative to the total imaging area between OAC(+) and OAC(−) in healthy and diseased eyes. Additionally, OAC(+) was applied to eyes with various retinal diseases as a pilot case study.

### 2.5. Statistical Analyses

For the white or black area in binarized images obtained using OAC(+) and OAC(−), we calculated the mean, standard deviation, and coefficient of variation (CV) across the studied population. Differences in the mean white (or black) area between the two methods were assessed using the paired *t*-test and Wilcoxon’s signed-rank test, and 95% confidence intervals were analyzed. All statistical analyses were conducted using JMP Pro 17.0.0 (JMP Statistical Discovery LLC, Cary, NC, USA) with a significance level of 5% (two-sided).

## 3. Results

### 3.1. Evaluation of OAC-Based EZ Assessment in Healthy Volunteer Eyes

**Figure 2:**
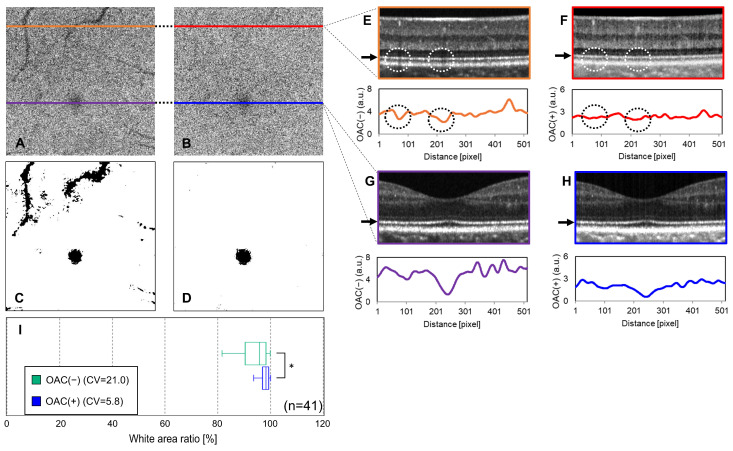
Comparison of the OAC-based method (OAC(+)) and intensity-based method (OAC(−)) in evaluating the EZ in healthy volunteer eyes. Panel (**A**) shows an en face image of the EZ band using the intensity-based method (OAC(−)), while panel (**B**) displays the image generated by the OAC-based method (OAC(+)). Panels (**C**,**D**) illustrate the binarized en face images for OAC(−) and OAC(+), respectively, highlighting reduced artifacts in the OAC(+) (**B**,**D**). B-scans are shown in (**E**,**G**) for OAC(−) and (**F**,**H**) for OAC(+), with arrows indicating the EZ depth in each B-scan. The B-scans also reveal diminished artifacts from retinal blood vessels with OAC(+) ((**E**,**F**), dashed circles). In both methods, the OCT signal in the fovea is notably attenuated in the B-scans (**G**,**H**), appearing as a black region in the binary images (**C**,**D**). The percentage of normal areas in the 41 healthy eyes was 96.2 ± 5.6% (CV = 5.8) for OAC(+), compared to 89.3 ± 18.8% (CV = 21.0) for OAC(−) ((**I**), paired *t*-test, * *p* < 0.05).

The study included 41 eyes from 41 healthy volunteers, including 21 men and 20 women with a mean age of 23.4 ± 2.4 years (21–34 years). The mean refractive error was −4.02 ± 2.64 D (−9.625 to +0.625 D). Representative images are presented in Figure 2. The OAC(−) en face image exhibited noticeable in-plane signal variation caused by projection artifacts (Figure 2A), which were mitigated in the OAC(+) en face image (Figure 2B). The binarized OAC(+) en face image (Figure 2D) effectively distinguished between hyper- and hypo-reflective regions and minimized projection artifacts, which were more pronounced in the binarized OAC(−) en face image (Figure 2C).

In the corresponding B-scan images, the EZ is depicted as a bright hyper-reflective band (black arrows in Figure 2E–H). The OCT attenuation and intensity profiles of each EZ slab are presented below the respective B-scan images (orange and purple: OAC(−); red and blue: OAC(+)). The OAC(−) B-scan image shows retinal blood vessels as projection artifacts, casting shadows and diminishing the EZ signal intensity (dashed circles in Figure 2E). Conversely, the OAC(+) B-scan image reduced these artifacts, resulting in a uniform profile (Figure 2F). The B-scan through the fovea revealed a flatter EZ line profile in the OAC(+) B-scan (Figure 2H) than in the OAC(−) B-scan (Figure 2G), although both methods consistently revealed a weaker signal in the fovea than in other regions. The estimated percent white area across the 41 healthy eyes was 96.2 ± 5.6% using OAC(+) versus 89.3 ± 18.8% using OAC(−) (Figure 2I and Table 1). The CV was 5.8% for OAC(+), indicating superior performance over OAC(−) (CV = 21.0%). A paired *t*-test confirmed that the mean white area obtained using OAC(+) was 6.9% greater than that obtained using OAC(−) (*p* = 0.036); this finding was supported by the Wilcoxon signed-rank test (*p* = 0.005).

### 3.2. Evaluation of OAC-Based EZ Assessment in Diseased Eyes

In total, 28 eyes from 34 participants with retinal diseases, including 16 male patients and 12 female patients with a mean age of 50.0 ± 17.0 years (14–78 years), were examined. These eyes included 25 cases of operated (previously fovea-off) retina detachments and three cases of central serous chorioretinopathy, and BCVA ranged 20/200–20/16.

**Figure 3:**
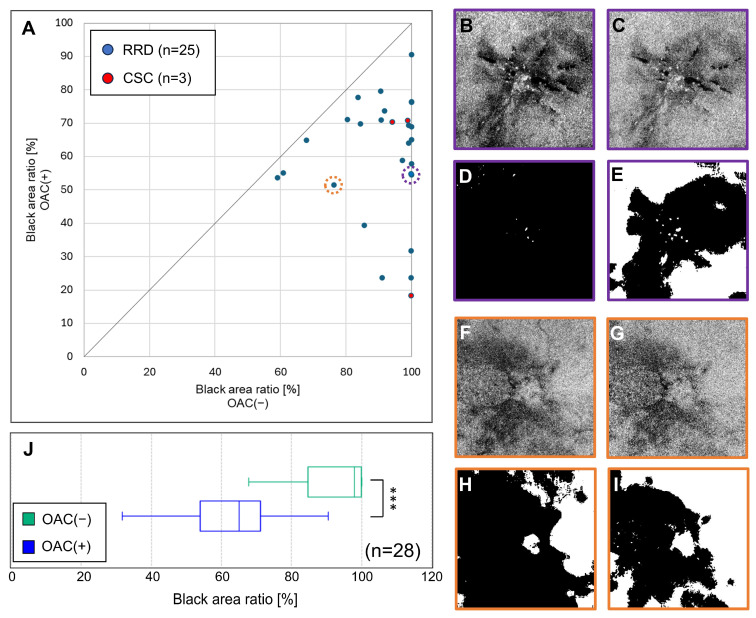
Comparison of the OAC-based method (OAC(+)) and intensity-based method (OAC(−)) in evaluating the EZ in diseased eyes. Panel (**A**) presents a scatter plot illustrating the ratio of the black area between OAC(+) and OAC(−). In OAC(−), most cases exhibited extensive black areas, indicating EZ impairments from moderate to complete EZ loss. The representative two cases of retinal detachment assessed using OAC(−) are presented in (purple circle = **B**, orange circle = **F**), whereas the assessments using OAC(+) are depicted in (**C**,**G**). Binarized OAC(−) images for case 1 ((**D**), black area = 99.9%) and case 2 ((**H**), black area = 76.2%) highlight the significant black areas. Conversely, the binarized OAC(+) images for cases 1 ((**E**), black area = 54.5%) and 2 ((**I**), black area = 51.4%) demonstrate reductions in the black area, indicating that OAC(+) accurately reflects the EZ states. (**J**) The estimated percent black area in diseased eyes (n = 28) was significantly lower with OAC(+) than with OAC(−) (60.1 ± 18.2% vs. 91.40 ± 12.2%, paired *t*-test, *** *p* < 0.001).

The scatter plot in Figure 3A indicates that the percent black area was ≥90% in 71.4% of cases using OAC(−). Conversely, the percent black area was ≥90% in 3.6% of cases with OAC(+), highlighting a substantial difference between these methods.

Examples of measurements with large and small differences in black areas between the two methods are indicated by purple and orange circles in the scatter plot. The first case involved a 28-year-old woman (BCVA = 20/40) who was assessed 4 months after buckling surgery for fovea-off retinal detachment (Figure 3B–E). The difference in the percent black area between the two methods reached 45.4%, with OAC(−) indicating a percent black area of 99.9%, thereby complicating EZ analysis. The second case involved a 39-year-old man (BCVA = 20/32) who was assessed 2 months after vitrectomy combined with encircling for proliferative vitreoretinopathy (Figure 3F–I). The difference in the black area between the methods was 24.7%, which was smaller than that in the aforementioned case.

In both cases, the OAC(−) en face images before binarization (Figure 3B,F) contained more artifacts than the OAC(+) images (Figure 3C,G), leading to substantial overestimation of the black area in the binarization results (Figure 3D,H). The binarized OAC(+) images (Figure 3E,I) more accurately reflect the EZ condition in both cases. Among the 28 eyes, the percentage of black area recorded by OAC(−) was 91.0 ± 12.2%, compared with 60.1 ± 18.2% for OAC(+), representing a significant difference between these methods (*p* < 0.0001 for both statistical tests, Figure 3J, Table 2).

### 3.3. OAC-Based EZ En Face Imaging for Various Retinal Disorders

**Figure 4:**
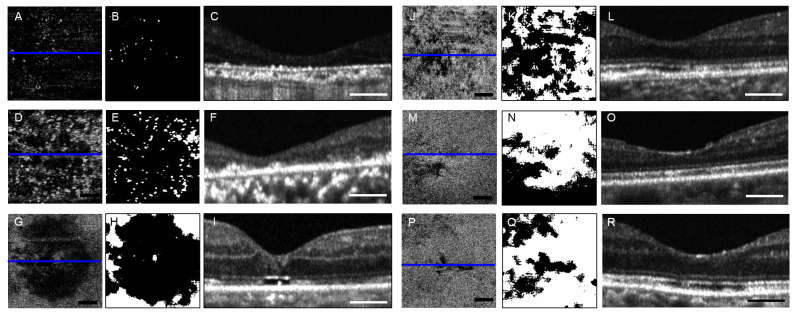
En face images and B-scan representations of EZ signals in six clinical cases analyzed using the OAC-based method (OAC(+)). Cases include Bietti crystalline chorioretinal dystrophy (**A**–**C**), rod–cone dystrophy (**D**–**F**), macular hole three months post-vitrectomy (**G**–**I**), chronic central serous chorioretinopathy without serous retinal detachment (**J**–**L**), occult macular dystrophy (**M**–**O**), and diabetic retinopathy following panretinal photocoagulation (**P**–**R**). Each condition is represented by three panels (e.g., (**A**–**C**)), with the left panel showing the en face images generated using OAC(+), the middle panel displaying the corresponding binarized images, and the right panel presenting the horizontal B-scan images of the foveal region(blue line). The black area in the binarized en face images represents EZ impairment, ranging from moderate to complete EZ loss, with the percentage of black area across these six cases ranging from 27.5% to 99.6%, with distinct patterns of white areas specific to each disease. The scale bar in each figure indicates 300 μm.

As a pilot study, we present examples of OAC(+) en face imaging in six diseased eyes in Figure 4. The details of these cases are provided in Table 3.

In all cases, the percentage of white area was smaller than that in healthy volunteers, and the patterns were diverse depending on the underlying pathology. The percent black area for the six patients was 27.5–99.6%. Among these cases, crystalline retinopathy (Figure 4A–C) and rod–cone dystrophy (Figure 4D–F) caused severe damage, characterized by significant retinal thinning and destruction of the outer layer structure, resulting in extremely large black areas (99.6% and 92.4%, respectively). Accurate segmentation of the EZ was not possible in such cases, suggesting that the EZ assessment was compromised. In a patient who underwent macular hole surgery, despite successful hole closure, B-scans indicated incomplete repair of the outer layer structure, revealing an extensive black area (76.4%), including within the fovea (Figure 4G–I). In a patient with chronic central serous chorioretinopathy after half-dose photodynamic therapy, patchy black areas accounted for 54.9% of the total area (Figure 4J–L). Notably, even in cases of occult macular dystrophy (Figure 4M–O) and diabetic retinopathy without macular edema (Figure 4P–R)—in which no significant abnormalities in the EZ structure were evident on B-scan images—the percent black areas were 41.6% and 27.5%, respectively.

## 4. Discussion

Herein, we developed a method to assess the EZ health in the macular region using a high-speed, high-resolution SS-OCT prototype. Our approach leverages OAC derived from raw OCT data to evaluate the EZ in the en face plane, representing a significant advancement over conventional OCT intensity-based methods. By applying this technique to healthy and diseased eyes, we demonstrated that EZ states can be visualized and quantified in a semiautomatic manner.

Previously, using commercially available SS-OCT to analyze EZ en face images following the repair of fovea-off retinal detachments reported interesting results [9]. This report noted the persistence of hypo-reflective regions in the EZ en face image within the detached region long after surgical intervention [9]. En face imaging provides a more comprehensive illustration of structural abnormalities than traditional assessments relying on EZ discontinuities on B-scan images. However, those previous intensity-based approaches often overlooked issues such as projection artifacts caused by retinal blood vessels, vitreous opacities, and other sources, which can obscure or distort EZ visualization and consequently affect the evaluation of the EZ band in the outer retina [9,20]. Our study successfully mitigated these artifacts using OAC instead of simply using signal intensity or its relative values, enabling a more selective assessment of the optical characteristics of the EZ band. Further, we addressed speckle noise using a nonlocal mean filter—a type of edge-preserving smoothing filter—and minimized motion artifacts through fast data acquisition using high-speed OCT, thereby enhancing the robustness of the measurements. Additionally, binarization using the Phansalkar method facilitated easier identification and quantification of structural abnormalities within EZ.

The evaluation in healthy eyes indicated that the CV was threefold smaller for OAC(+) than for OAC(−) (Figure 2, Table 1). Statistical analyses confirmed that the mean percent white area measured by OAC(+) was significantly larger than that obtained using OAC(−) (Table 1). In healthy eyes, a predominance of white area is expected unless distorted by projection artifacts, and this result was consistently reproduced by the OAC(+) method. This highlights the reliability of OAC as a surrogate marker of EZ states.

In diseased eyes, OAC(+) revealed a significant reduction in the percentage of black area. Although the primary advantage of OAC lies in its artifact-reduction capabilities, they normalize values across imaging sessions because OAC is a physical quantity. This robustness against variations in imaging conditions enhances its utility in monitoring retinal health and disease progression. This normalization potentially reduced binarization errors among diseased eyes (Figure 3, Table 2).

Figure 4 illustrates that binarization using the Phansalkar method permitted quantification of the extent of EZ damage across various retinal disorders. Across retinal disorders, en face images obtained using OAC(+) effectively revealed areas of structural abnormality in the EZ compared with evaluations using B-scan images alone. However, EZ segmentation is not meaningful in severe cases involving extensive damage in outer retinal structures (Figure 4A–F). Although we defined the EZ slab based on the residual EZ fragments, making a correct evaluation was difficult. Even if we exclude these two cases, the black regions present in diseased eyes considerably represent a spectrum of conditions, including irrecoverable damage, regions with recovering function but structural abnormalities, and other variably affected regions [21] (Figure 4G–R). Distinguishing these regions remains a significant challenge. Thus, establishing appropriate thresholding is crucial for enhancing sensitivity and specificity in disease screening and monitoring.

## 5. Limitations

This study had several limitations. First, the primary goal of establishing the measurement program limited our evaluation to small numbers of actual clinical cases. Future investigations should explore the relationship between EZ abnormalities and visual function, including BCVA, to enhance its clinical applicability. Clinical validation with large sample sizes and longitudinal studies will also be crucial for assessing changes over time. In addition, we would like to consider using this program to visualize and evaluate diseases such as reticular pseudodrusen (RPD), geographic atrophy (GA), and other diseases that could not be shown in this study. Second, although OAC provides valuable insights, it does not completely describe the EZ condition. Factors such as the Stiles–Crawford effect [22,23,24], anatomical features of the foveal bulge, and structural characteristics of foveal cone photoreceptors—which exhibit minimal taper compared with peripheral cones [25]—can influence OAC calculations. The darker region frequently observed around the fovea (Figure 2C,D) reflects these effects. Future studies should aim to refine OAC calculation methods to account for light-coupling efficiency or directional-dependent scattering effect [26]. Additionally, the implications of multiple scattering, absorption, confocal function, and other variables warrant exploration to enhance the accuracy of OAC calculations [27,28].

## 6. Conclusions

The OAC-based method for EZ visualization and quantification offers significant advantages over traditional intensity-based approaches, including reduced artifacts and increased reliability. This method holds promise for improving the understanding of pathologies by physicians and patients by providing accurate and visually understandable results regarding EZ conditions. Future research should address the method limitations and expand its application to validate its clinical utility.

## Figures and Tables

**Table 1:** White areas for normal eyes with and without OAC-based analysis (n = 41).

	Mean (%)	SD	Min	Max	CV
OAC(+)	96.2	5.6	77.2	100	5.8
OAC(−)	89.3	18.8	0.5	99.8	21.0
	Mean difference	SE	95% CI	*p*-value ^1^	*p*-value ^2^
	6.9	3.2	[0.5, 13.3]	0.036	0.005

Abbreviations: OAC: optic attenuation coefficient, SD: standard deviation, CV: coefficient of variation, SE: standard error, CI: confidence interval. ^1^: paired *t*-test, ^2^: Wilcoxon’s signed-rank sum test.

**Table 2:** Black areas in diseased eyes with and without OAC-based analysis (n = 28).

	Mean (%)	SD	Min	Max	
OAC(+)	60.1	18.2	18.2	90.5	
OAC(−)	91.0	12.2	59.1	100	
	Mean difference	SE	95% CI	*p*-value ^1^	*p*-value ^2^
	−31.0	4.1	[−39.5, −22.5]	<0.0001	<0.0001

Abbreviations: OAC: optic attenuation coefficient, SD: standard deviation, SE: standard error, CI: confidence interval. ^1^: paired *t*-test, ^2^: Wilcoxon’s signed-rank sum test.

**Table 3:** Clinical characteristics of patients who underwent OAC-based analysis.

Pt	Diagnosis	Sex	Age	Eye	BCVA	Black Area (%)	Therapeutic Intervention
1	Bietti crystalline chorioretinal dystrophy	M	62	OS	20/50	99.6	None
2	Rod–cone dystrophy	F	43	OD	20/50	92.4	None
3	Macular hole	M	74	OS	20/100	76.4	20 days after vitrectomy
4	Chronic central serous chorioretinopathy	M	46	OS	20/25	54.9	31 months after half-dose PDT
5	Occult macular dystrophy	M	79	OS	20/100	41.6	None
6	Diabetic retinopathy	F	59	OD	20/32	27.5	After panretinal photocoagulation

Abbreviations: OAC: optic attenuation coefficient, BCVA: best-corrected visual acuity, OS: left eye, OD: right eye, PDT: photodynamic therapy.

## Data Availability

The data presented in this study including extracting data from patient records are available on request from the corresponding author.
